# Supplementary material for: A comparison of match outcomes between traditional medical degree and dual-degree applicants
Source: PLoS One. 2020 Dec 18;15(12):e0244147. doi: 10.1371/journal.pone.0244147 (PMC7748145; doi:10.1371/journal.pone.0244147)
Supplement: Attachment — Submitted filename: Response letter to PLoS ONE.docx [file pone.0244147.s002.docx]

Editors of PLoS ONE

November 25, 2020

Dear Sirs:

Thank you for allowing us to resubmit our manuscript. We will address each of the reviewer’s comments as follows:

a) **If there are ethical or legal restrictions on sharing a de-identified data set, please explain them in detail (e.g., data contain potentially identifying or sensitive patient information) and who has imposed them (e.g., an ethics committee). Please also provide contact information for a data access committee, ethics committee, or other institutional body to which data requests may be sent.**

The data is from the publicly available Texas STAR database. We have attached a sample data set used for this study.

b**) If there are no restrictions, please upload the minimal anonymized data set necessary to replicate your study findings as either Supporting Information files or to a stable, public repository and provide us with the relevant URLs, DOIs, or accession numbers. Please see** [**http://www.bmj.com/content/340/bmj.c181.long**](http://www.bmj.com/content/340/bmj.c181.long) **for guidelines on how to de-identify and prepare clinical data for publication. For a list of acceptable repositories, please see** [**http://journals.plos.org/plosone/s/data-availability#loc-recommended-repositories**](http://journals.plos.org/plosone/s/data-availability#loc-recommended-repositories)**.**

**We will update your Data Availability statement on your behalf to reflect the information you provide.**

We will make the required upload.

3. **PLOS requires an ORCID iD for the corresponding author in Editorial Manager on papers submitted after December 6th, 2016. Please ensure that you have an ORCID iD and that it is validated in Editorial Manager. To do this, go to ‘Update my Information’ (in the upper left-hand corner of the main menu), and click on the Fetch/Validate link next to the ORCID field. This will take you to the ORCID site and allow you to create a new iD or authenticate a pre-existing iD in Editorial Manager. Please see the following video for instructions on linking an ORCID iD to your Editorial Manager account:** [**https://www.youtube.com/watch?v=_xcclfuvtxQ**](https://www.youtube.com/watch?v=_xcclfuvtxQ)

We will make the requested update. The corresponding author’s ORCHID iD is: 0000-0002-9781-9316

**Reviewer #1: This study presents residents data from over 100 institutions to examine the characteristics of residents with MD only vs dual degree MD and study the relationship of these degrees with match outcomes. This study is trying to fill the knowledge gap in match competivity by analyzing the data with additional degrees among MDs. There are several methodological and statistical issues that need to be addressed in this study.**

We thank the reviewer for their comments.

**Please specify the range of years (though this was specified in abstract and conclusion) that the data was achieved from STAR database and the total number of residents when describing your data. It is not clear, if the investigators used the aggregate data or resident level individual data in this study. If they have used an aggregate level data, ecological fallacy might exist and need to account for that. The clarity on the STAR database need to be elaborated together with the types of variables used.**

We added text to specify the years of the study data (2017-2019). The total number of residents is also included (18,224). We additionally clarified in the manuscript that this study used individual resident level data, not aggregate data.

**The authors have used two-sample t-test to compare means. T-test is a parametric approach. Given the sample size, the central limit theorem might be applicable here. However, it is important to check the assumptions of the parametric approaches before applying t-tests.**

We chose a simple t-test as is easily understood and widely used. Additionally, the variables studied are assumed to be parametric. As an example, the probability characteristics of USMLE scores are distributed according to a normal distribution by design. Because the probability distribution is defined, a parametric method (like the Student’s t-test) is appropriate. Similarly, our other variables are assumed parametric.

**Please specify the variables that were treated as continuous and those as categorical. There are some discrete variables, for example, programs applied, interviews attended. Treating these as continuous and using t-test might not be correct due to the skewness of the data. Please confirm and provide justification to treat these as continuous variables to apply t-tests.**

Continuous variables included USMLE scores, honored clerkships, research experiences, programs applied to, interview number and most of the variables in Table 3. Because of the large number of students in the data set (over 18,000) we assumed lack of skewness and a normal distribution.

**In chi-square tests of proportions, some of the expected proportions are very small (with expected cell count <5). The chi-square tests will not be applicable for such small cell counts; however, you might want to check the significance tests using some exact methods (e.g. Fisher’s exact test).**

None of the cells had less than a count of 5. Chi-squared analysis is typically used in such comparisons.

**Multiple pairwise comparisons are done here using MD alone as the comparison group. Were p-values accounted for these multiple testing? Please comment.**

P-values were accounted for multiple pairwise testing as suggested.

**Since no significant difference was seen by degree types, please comment of the power of the tests to rule out any Type II errors.**

The p-values in Table 1 suggest that we can exclude the null hypothesis with less than 95% certainty. For studies of this type, this is typically represented as the groups not being statistically different. Since our study included over 17,000 participants, we feel that the study has the power to rule out Type II errors.

**The primary aim of this study was to compare the match rates by single vs dual degrees. It seems that there are several applicant characteristics that are significantly different between the degrees. These characteristics in addition to some baseline characteristics (such as age, gender, race/ethnicity, interest in area of medicine, etc. if any) might confound the relationship between the degrees and match rates. Did the authors see the relationship between the degrees and match rates in multivariable analysis?**

Although interesting questions, the database does not include the additional information suggested by the reviewer. Further, as the data submitted is blinded, additional demographic information could make the data more identifiable. As such, it is not collected by Texas STAR.

**Did the difference (no difference) exist by the school type (private, public)? Suggestion: You can analyze this by stratified analysis using school types as stratifying variables**.

This is an interesting question but not one addressed by our current study.

**I totally understand the feasibility of the length/width of the table to present the actual p-values instead of p<0.05. However, it would be helpful to understand the strength of association is raw p-value is presented as supplement.**

Actual p-values are presented in Table 1.

**Since the study is voluntary and survey-based, the missing data might be of another limitation. Please comment on the range of item missing rates. Also comment on what strategies did you use to account for the missing data?**

We describe missing data in our discussion. Missing data is impossible to collect as participation in the Texas STAR database is voluntary.

We thank the reviewers for their thoughtful comments.

Sincerely,

Marc J. Kahn, MD, MBA

Dean and Professor of Medicine

UNLV School of Medicine
